# Relapsing Polychondritis: A Case of Marked Improvement After Steroid Administration for Airway Obstruction

**DOI:** 10.7759/cureus.51101

**Published:** 2023-12-26

**Authors:** Takanori Ohno, Ayako Shimada, Yuuko Terada, Toshitaka Ito, Kazuyuki Miyamoto

**Affiliations:** 1 Department of Emergency Medicine, Shin-Yurigaoka General Hospital, Kawasaki, JPN; 2 Department of Respiratory Medicine, Shin-Yurigaoka General Hospital, Kawasaki, JPN; 3 Department of Emergency and Disaster Medicine, Showa University, Tokyo, JPN

**Keywords:** relapsing polychondritis, hydrocortisone, saber-sheath, tracheostenosis, asphyxia

## Abstract

Relapsing polychondritis is a rare disease that causes progressive and recurrent destruction of cartilage in the auricles, eyes, nose, and airways. A 90-year-old man was brought to the emergency department with fever, low SpO_2_, and effortful breathing. Arterial blood gas analysis showed that PaCO_2_ levels had accumulated to 120 mmHg. Although CT showed marked thickening of the bronchial wall from the central to the peripheral region, the cause was unknown. At the family's request, the patient was not placed on a ventilator, and treatment was started with steroids alone. After admission, the patient’s condition improved with only intravenous steroids, and he was discharged to the facility with continued oral steroid medication. After a short treatment period, the possibility of relapsing polychondritis was considered and confirmed. The patient met Levine's diagnostic criteria, with findings of destruction of the bilateral auricular cartilage and the airway and a response to steroid administration. Although it is very difficult to diagnose relapsing polychondritis at the initial emergency department visit, early administration of steroids is worth trying in patients with asphyxia with extensive thickening of the airway on CT findings, as relapsing polychondritis may be considered, and early steroid administration may improve patient symptoms.

## Introduction

Relapsing polychondritis is most common in middle-aged individuals (typically 40-55 years) and is observed in all age groups [1]. It is slightly more common in women, with a male-to-female ratio of 1:1.8, according to a survey of 200 patients reported in the literature [2]. The condition occurs in individuals of all races but tends to be more common in Caucasians [3]. The disease is characterized by recurrent inflammation and progressive destruction of cartilage of unknown causes. The affected cartilages include the auricle, eyes, nose, joints, and airways, where recurrent inflammatory reactions occur. In addition to the respiratory tract, it can occur less frequently in the heart and central nervous system. Although early detection is important, diagnosis is based mainly on physical examination, as there are no characteristic biomarkers, such as blood tests or imaging.

## Case presentation

A 90-year-old man residing in an elderly care facility had a history of early gastric cancer (untreated), prostate cancer, dementia, hypertension, and heart failure and was taking amlodipine besylate and furosemide. Although he had dementia, he had no difficulty in his daily life with assistance. When the staff checked on the patient late at night, sputum clusters, fever, and a drop in SpO_2_ were observed, which led to a call for emergency medical assistance. During EMS contact, he had a respiratory rate of 24/min, SpO_2_ of 60%, BP of 151/84 mmHg, HR of 128/min, GCS of E4V4M6, and BT of 38.1 ℃.

**Figure 1:**
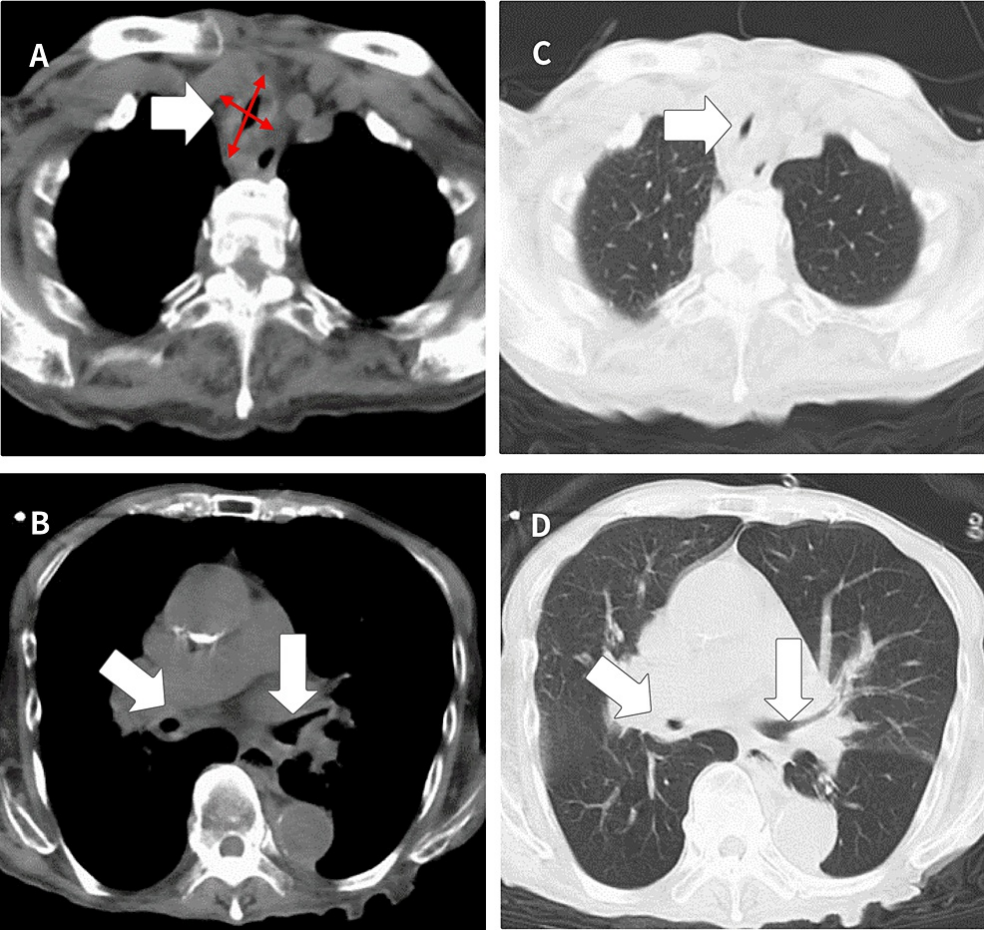
CT: (A and B): Soft tissue imaging conditions and (C and D): lung field imaging conditions. As indicated by the white arrows, there was a thickening of the bronchial wall from the central to the peripheral region. On the central side (A, C), the anteroposterior diameter is longer than the transverse diameter (red arrows) due to the lack of wall thickening in the membranous part of the posterior surface, resulting in a saber-sheath-like morphology. No morphological changes were seen in the bronchi after the bifurcation because of the absence of the membranous part of the posterior wall.

On arrival at the hospital, his respiratory rate was 22/min, SpO_2_ 38%, BP 147/83 mmHg, HR 71/min, and BT 39.0 ℃. The patient was breathing effortfully, and oxygen was temporarily turned off following blood gas collection; however, bradycardia progressed gradually, and the patient temporarily stopped breathing. Arterial blood gas analyses showed pH 7.122, PaO_2_ 28.4, PaCO_2_ 118.9, HCO_3_ 37.2, BE 5.7, and Lac 1.1, indicating a marked respiratory acidosis with carbon dioxide retention (other blood tests are shown in Table 1). The patient had a history of hypertension and heart failure but no asthma, chronic obstructive respiratory failure, anaphylaxis, or other allergic-like symptoms. On physical examination, wheezing was heard at the periphery of the bilateral lung fields, but no stenotic sounds were heard in the central airway. Simple CT showed no evidence of pneumonia or congestion and extensive thickening of the bronchial wall from the main bronchi to the obliterated bronchioles, which was thought to be the cause of alveolar hypoventilation (Figure 1).

**Table 1:** Laboratory data at the time of hospital arrival. No increase in Krebs von den Lungen-6 or Surfactant protein D, which indicates the degree of lung damage, and the impact of interstitial pneumonia was judged to be low. White blood cell and C-reactive protein were elevated, but procalcitonin was not. IgE was particularly elevated in immunoglobulins.

Complete blood counts		
White blood cells (/µL)	13900	3300-8600
Erythrocytes (×10^4^/μL)	290	435-555
Hemoglobin (g/dL)	9.1	13.7-16.8
Hematocrit (%)	29.4	40.7-50.1
Platelets (×10^4^/μL)	60.1	15.8-34.8
Biochemical parameters		
Krebs von den Lungen-6 (U/mL)	116	500>
Surfactant protein D (ng/mL)	<15.0	110.0>
C-reactive protein (mg/dL)	12.79	0.3>
Procalcitonin (ng/dL)	0.15	0.5>
IgG (mg/dL)	1463	870-1700
IgG4 (mg/dL)	92.4	11.0-121.0
IgA (mg/dL)	432	110-410
IgM (mg/dL)	139	33-190
IgE (IU/dL)	1325	170>
Rheumatoid factor (IU/mL)	7	15>
Arterial blood gases (ambient air)		
pH	7.122	7.35-7.45
PaCO_2_ (mmHg)	118.9	35-45
PaO_2_ (mmHg)	28.4	80-100
HCO_3_^-^ (mmol/L)	37.2	22-26
Base excess (mmol/L)	5.7	-2-2
Lactic acid (mmol/L)	1.1	0.5-2.2

The patient had an obstruction due to extensive bronchial wall thickening, which resulted in carbon dioxide retention. Although ventilatory management was appropriate owing to the extensive obstruction, the family did not wish to introduce a ventilator; therefore, no other measures were taken except for oxygen administration.

Although there was no hope of prolonging his life, the patient was treated with infusions. We considered the possibility of autoimmune disease but were not sure at this point, so we administered 0.3 mg of inhaled beta stimulant and 200 mg intravenously as per asthma treatment. Because the patient also had a fever, a blood culture was performed, and azithromycin (500 mg) was administered in consideration of diffuse bronchiolitis. However, the blood culture results were negative.

**Figure 2:**
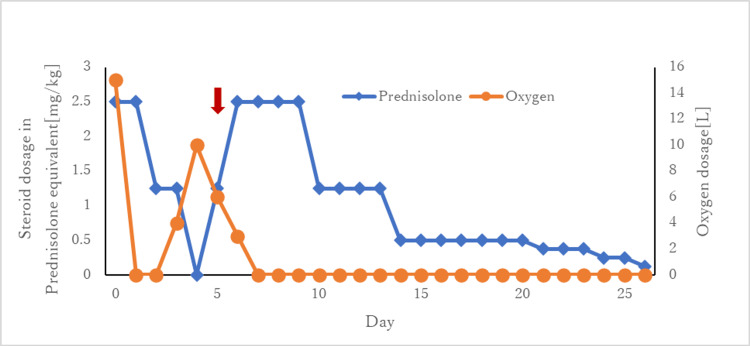
Changes in steroid and oxygen dosages. The administered steroids are converted to weight in prednisone equivalents and are expressed in doses per body weight. After admission, oxygen demand improved, and steroid administration was rapidly tapered off, yet oxygen demand increased again. When steroids were resumed at a dose derived from a prior study, oxygen demand improved, and the dose was carefully tapered off thereafter. The red arrows indicate relapsing polychondritis, which was suspected based on the improvement of symptoms in steroid dependence, and again to check for other initial cartilage findings.

**Figure 3:**
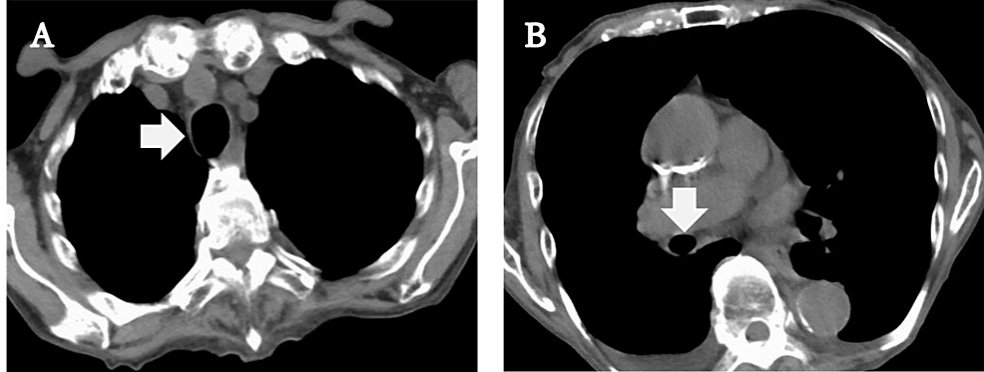
Post-treatment plain CT. A is the central side, and B is the peripheral side from the bifurcation. Bronchial wall thickening had disappeared. The central bronchus, which had been lengthened in the anterior-posterior direction, was also improved and became a round form.

After admission, the patient continued to receive 40 mg methylprednisolone intravenously twice daily, and the antibiotics were changed to ceftriaxone (1 g) once daily. Nine hours after the first steroid dose, the patient was able to speak. On the fourth day, the steroids were discontinued; however, the patient's oxygenation worsened again, so the steroids were continued. Finally, the steroid was changed from intravenous infusion to oral medication, and the patient was discharged from the hospital after adjusting for 5 mg of prednisone. The changes in steroid administration and oxygen demand are shown in Figure 2. A plain CT taken before discharge showed improvement in bronchial wall thickening (Figure 3).

## Discussion

Relapsing polychondritis is a very rare disease, with a reported incidence of 0.7 to 9.0 cases per million people per year [4] and only approximately 500 cases in Japan [5]. In the past, there have been reports of patients with cardiopulmonary arrest due to asphyxia caused by airway narrowing, similar to the present patient; however, even in those cases, the diagnosis was not made at the initial presentation [6].

Inflammation of the auricular (70%), joint (36%), and nasal (26%) areas is a common clinical manifestation [3]; in severe cases, asphyxia, similar to the present patient, occurs in the airway (21-56%), and aortic and mitral valve insufficiency due to valve ring dilation occurs in the heart and vascular system (6-27%) [1]; therefore, early diagnosis is necessary.

The physical characteristics of the disease are characterized by recurrence and destruction of cartilage in the auricular and nasal root areas, and diagnosis is relatively easy when a specialist physician examines the patient. However, due to the lack of awareness of the disease, it has been reported to take 1.9 to 10 years from onset to diagnosis, with an average of five clinician visits [3].

The first set of diagnostic criteria was compiled by McAdam et al. [7] in 1976, followed by Damiani and Levine in 1979 [8], who added McAdam's criteria for histological findings and treatment effects. The most recent version was published by Michet et al. in 1986 (Table 2) [9,10]. These criteria are used for diagnosis, which requires initial inflammatory findings in multiple locations.

**Table 2:** Diagnostic criteria for relapsing polychondritis.

	McAdam’s criteria (1976)	Damiani and Levine’s criteria (1979)	Michet’s criteria (1986)
Symptoms	1. Bilateral auricular chondritis, 2. Non-erosive seronegative inflammatory arthritis, 3. Nasal chondritis, 4. Ocular inflammation, 5. Respiratory tract chondritis, 6. Audiovestibular damage	Criteria A: 1. Bilateral auricular chondritis, 2. Non-erosive seronegative inflammatory arthritis, 3. Nasal chondritis, 4. Ocular inflammation, 5. Respiratory tract chondritis, 6. Audiovestibular damage	Major criteria: 1. Auricular chondritis, 2. Nasal chondritis, 3. Laryngotracheal chondritis
			Minor criteria: 1. Ocular inflammation (conjunctivitis, episcleritis, uveitis, keratitis), 2. Hearing loss, 3. Vestibular dysfunction, 4. Seronegative polyarthritis
		Criteria B: Positive biopsy	
		Criteria C: Positive response to glucocorticoids or dapsone	
Required criteria	≥ 3	≥ 3 of Criteria A or ≥ 1 of Criteria A and Criteria B or ≥ 2 of Criteria A and Criteria C	≥ 2 Major criteria or ≥ 1 Major criteria and 2 Minor criteria

Laboratory findings were primarily used to exclude ANCA-related diseases. While certain anti-type II collagen antibodies have been reported to be effective [11], others have shown no sensitivity or specificity [1], making them only adjunctive diagnostics. Diseases that cause bronchial wall deformities and mucosal thickening on CT include relapsing polychondritis, amyloidosis, and granulomatosis with polyangiitis. The difference between relapsing polychondritis and other diseases is characterized by a saber-sheath-like morphology due to the lack of thickening of the posterior wall of the trachea, where no cartilage is present [12]. Notably, this finding is very difficult to identify in the emergency department without prior knowledge. Recently, FDG-PET has also been reported to be effective [13], although it is not yet widely used. In this case, the patient was unable to undergo bronchoscopy in the acute stage; therefore, a tissue diagnosis could not be performed. However, there is disagreement regarding whether tissue diagnosis should be performed in the acute stage because of the risk of infection, airway stenosis, and obstruction [14]. To make a definitive diagnosis, it was necessary to identify multiple physical findings; however, we missed the necessary physical findings at the initial stage due to the lack of awareness of the disease.

**Figure 4:**
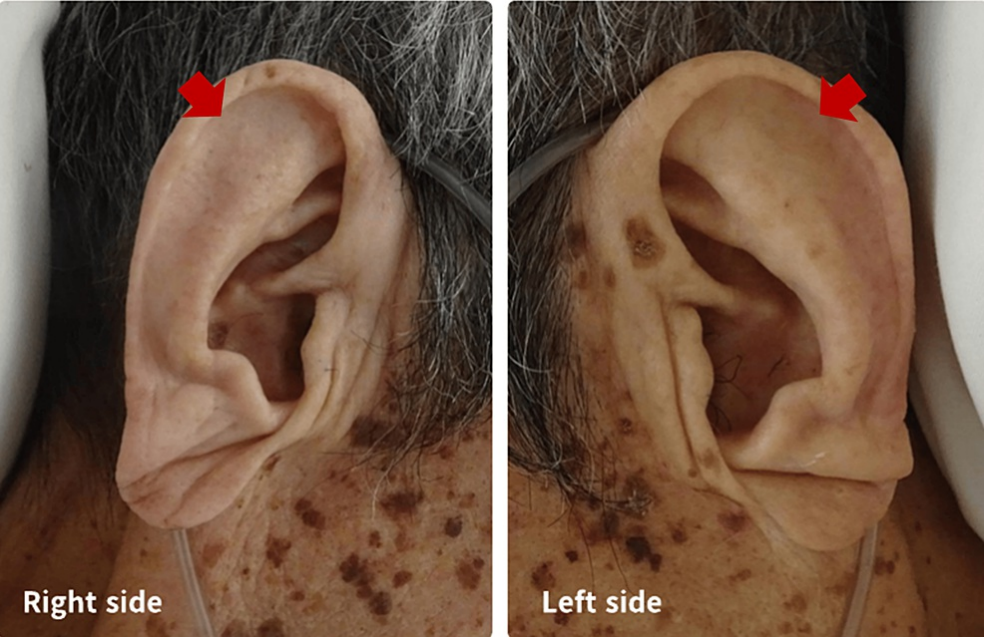
Findings of bilateral auricles. There was no obvious redness due to post-steroid administration; however, the bilateral auricles were deformed and the counter-auricles were missing (red arrow).

In this case, the patient had marked respiratory acidosis when he first arrived at the hospital, but his family did not wish to use a ventilator; therefore, treatment was started without respiratory support. A simple CT revealed extensive thickening of the bronchial wall, but no causative disease could be identified, and from the results of the search, we considered IgG4-related diseases [15] and other autoimmune diseases and administered hydrocortisone 200 mg. The response to steroids was good, and the patient improved without the use of a ventilator. Although the patient responded to steroid treatment, examination of other parts of the body revealed a deformity of the auricular cartilage (Figure 4), which met Damiani and Levine's diagnostic criteria. Histological examination of the auricular cartilage was not performed because the patient had already started steroid treatment, and his condition was already stable.

As a result, the early administration of steroids in the emergency department led to a successful outcome, which may be because it occurred before tracheal cartilage destruction. If a diagnosis of relapsing polychondritis is made at the initial treatment stage, methylprednisolone (1000 mg/day for 1-3 days) is recommended, depending on the severity of the disease. In the most severe cases, high-dose steroids plus immunosuppressive drugs are recommended [1]. There have been reports on the use of biological agents in patients with relapsing polychondritis to conventional therapy [16]; however, since they may not be covered by medical insurance, their use should be performed with caution. As for the respirator, its use was also an option, although it would not be a life-prolonging treatment. It is recommended that oral steroids be continued at a dose of prednisone not exceeding 60-70 mg/day for at least three weeks, after which the dose should be tapered and adjusted to less than 15 mg/day after three months and less than 10 mg/day after six months [1].

It is difficult to diagnose and initiate treatment for relapsing polychondritis in the emergency department. However, the disease that should be considered when characteristic thickening of the bronchial wall is observed on imaging, in addition to respiratory acidosis associated with asphyxia. For such patients, we believe that steroid administration should be considered early, in addition to the usual resuscitation procedures, even if a diagnosis is not made.

## Conclusions

Relapsing polychondritis carries a risk of death when associated with airway narrowing. Therefore, there have been reports of patients who went unnoticed at the initial presentation, and the treatments were not life-saving. Usual respiratory management alone is insufficient, and the use of steroids or immunosuppressive drugs must also be considered for this disease.

In contrast, in patients with asphyxia due to thickening of the bronchial wall, even if a diagnosis cannot be made in the emergency department, suspicion of autoimmune disease and steroid administration may be sufficient to save the patient's life. Therefore, in patients with airway obstruction associated with relapsing polychondritis, even if the diagnosis is not made at the time of transport, it is important to resuscitate the patient first, check the response to a small dose of steroids, and then, after admission, confirm the findings at other cartilage sites and compare them with the diagnostic criteria.

.
